# Immunohistochemical Studies on Galectin Expression in Colectomised Patients with Ulcerative Colitis

**DOI:** 10.1155/2016/5989128

**Published:** 2016-01-18

**Authors:** Mattias Block, Johan Mölne, Hakon Leffler, Lars Börjesson, Michael E. Breimer

**Affiliations:** ^1^Department of Surgery, Sahlgrenska Academy, University of Gothenburg, Sweden; ^2^Department of Pathology, Sahlgrenska Academy, University of Gothenburg, Sweden; ^3^Department of Laboratory Medicine, Lund University Hospital, Lund, Sweden

## Abstract

*Introduction*. The aetiology and pathogenesis of ulcerative colitis (UC) are essentially unknown. Galectins are carbohydrate-binding lectins involved in a large number of physiological and pathophysiological processes. Little is known about the role of galectins in human UC. In this immunohistochemical exploratory study, both epithelial and inflammatory cell galectin expression were studied in patients with a thoroughly documented clinical history and were correlated with inflammatory activity.* Material and Methods*. Surgical whole intestinal wall colon specimens from UC patients (*n* = 22) and controls (*n* = 10) were studied. Clinical history, pharmacological treatment, and modified Mayo-score were recorded. Tissue inflammation was graded, and sections were stained with antibodies recognizing galectin-1, galectin-2, galectin-3, and galectin-4.* Results*. Galectin-1 was undetectable in normal and UC colonic epithelium, while galectin-2, galectin-3, and galectin-4 were strongly expressed. A tendency towards diminished epithelial expression with increased inflammatory grade for galectin-2, galectin-3, and galectin-4 was also found. In the inflammatory cells, a strong expression of galectin-2 and a weak expression of galectin-3 were seen. No clear-cut correlation between epithelial galectin expression and severity of the disease was found.* Conclusion*. Galectin expression in patients with UC seems to be more dependent on disease focality and individual variation than on degree of tissue inflammation.

## 1. Introduction

Ulcerative colitis (UC) is a chronic, relapsing, and remitting inflammation of the large intestine [1]. It often starts at young age and lasts throughout life. Debilitating symptoms, such as increased frequency of bloody stools, pain, fever, and lack of effective treatment, lead to the need for surgical removal of the whole large intestine in one-third of the patients [2]. For these reasons it is of great interest to understand the molecular mechanism behind the disease in order to identify new targets that can be modified as therapy. The aetiology and pathogenesis of UC are unclear [3] but are known to include the intestinal microflora, the intestinal barrier function, and the immune system and modifications of these by genetic polymorphisms [4].

**Figure 1:**
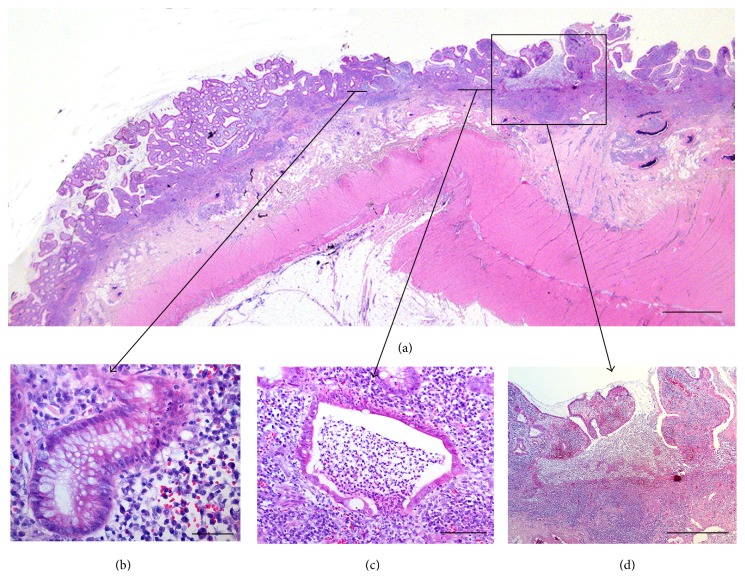
Illustration of the histological features for inflammatory grading of UC colon mucosa according to [17]. Top panel (a) shows a haematoxylin-eosin stained colon section from a patient with severe inflammatory activity (Table 1, patient 11, left colon). Below are shown magnifications of three selected areas illustrating cryptitis (panel (b), marked with black arrows), abscess (c), and an ulceration (d) all present in close proximity to each other. Bar in (a) = 2 mm, (b) = 50 *μ*m, (c) = 100 *μ*m, and (d) = 1 mm. Note that the sizes of biopsies obtained by endoscopy usually are 3-4 mm.

Galectins, a family of soluble carbohydrate-binding proteins (lectins), have emerged as one possible therapeutic target in inflammatory bowel disease (IBD). This is based mainly on experiments in animals and cell culture [5] pointing to potential pathophysiology roles in IBD, the fact that galectins tend to be well tolerable, and the therapeutic effects of galectin inhibitors in inflammatory disease of other tissues. Galectins are defined by a conserved carbohydrate recognition domain (CRD) with affinity for *β*-galactosides as found in glycoproteins and glycolipids [6] and occur in different types as shown in Figure 1. Galectins are synthesized in the cytosol and may have functions there and in the nucleus [7].

An important emerging mechanism of action involves their transfer, by nonclassical secretion, into vesicles or extracellularly, where they encounter *β*-galactoside containing glycoproteins, which they may cross-link. This enables galectins to direct subcellular trafficking, organize membrane architecture, affect cell adhesion, and/or induce cell signals in the same or other cells [7–9]. This in turn is manifested at the organism level as rate-limiting effects on inflammation, immunity, and cell growth [10–12]. In the large intestine, additional potential galectin mediated effects exist including modification of barrier function and interaction with microbes [13]. For this reason, galectins represent targets for therapeutic intervention of disease [14–16].

Various aspects for the role of galectins in IBD pathogenesis have been studied, most of these in mouse colitis models [5]. In these models, both pro- and anti-inflammatory properties of different galectins have been identified [18–24] and related to concomitant changes in glycan structures [22, 23]. Galectin-1 is mainly anti-inflammatory, by induction of apoptosis in T-cells [5]. Galectin-2 has been studied much less but has been found mainly to be anti-inflammatory and supporting wound healing in the intestine [19]. Galectin-3 has been studied extensively and it is required for polarized targeting of some glycoproteins in intestinal epithelial cells [7, 24]. It is also highly expressed in macrophages and is mainly proinflammatory but also protects against tissue damage. Recent studies suggest rate-limiting roles of galectin-3 in the fibrosis accompanying some chronic inflammation [25]. The role of these different effects in intestinal inflammation remains unclear [5]. Galectin-4 is highly and specifically expressed in intestinal epithelial cells and may direct polarized trafficking [26] and formation of super rafts [27] as well as being bactericidal [28]. In IBD, galectin-4 has the most distinct pathogenic role as a specific activator of intestinal, but not other, CD4-positive T-cells [29].

Galectins have been studied in human IBD both as serum biomarkers [30] and in tissue samples, but there are restrictions in the clinical interpretation of data obtained since patient classification in terms of disease severity, intestinal inflammatory grade, and pharmacological treatment is limited [31–33]. Most studies were performed using tissue biopsies obtained by endoscopy that only allow evaluation of a limited amount of superficial mucosal tissue from selected areas of the intestinal wall. Considering the complexity of UC, the aim of this study was to explore the galectin expression in whole intestinal wall surgical specimens, not earlier performed, as well as correlate epithelial galectin-1, galectin-2, galectin-3, and galectin-4 expression with degree of intestinal inflammation.

## 2. Material and Methods

### 2.1. Patients

All patients with IBD undergoing acute or elective colectomy, proctocolectomy, or rectal resection at the Colorectal Unit, Sahlgrenska University Hospital/Östra Campus, from 2008 to 2011 were, prior to surgery, asked to participate in a multidisciplinary IBD research project approved by the Regional Ethical Review Board, University of Gothenburg, Sweden (http://www.epn.se). Among 78 included IBD patients, 22 consecutive patients with UC disease were included in this study and patient data is listed in Table 1. Excluded patients were those with UC operated with extirpation of ileal pouch-anal anastomosis (IPAA) and those with Crohn's disease. In addition, some patients had to be excluded due to logistic reasons such as lack of laboratory staff and out-of-office-hour surgery. Prior to surgery, all patients were examined and categorized using a modified Mayo-score [34] to measure disease severity with a scale range of 0–12, 12 being most severe disease. Variables measured were frequency of bowel movements (range 0–3), blood in stool (range 0–3), quality of life (range 0–3), and endoscopic evaluation (range 0–3). Only two patients undergoing acute resection were included in this study (cases 3 and 28). Ongoing and previous medications, in particular the use of steroids, 5-ASA, and immunomodulators such as TNF inhibitors, were listed. Median age of included patients was 38 (20–65). Median laboratory tests were haemoglobin 136 (101–156) g/L, C-reactive protein (CRP) 5 (1–160) mg/L, and albumin 38 (20–46) g/L. Symptom duration was 8 (2–25) years. Total modified Mayo-score was median 8 (5–11) and there was no correlation between the Mayo-score and the histopathological inflammatory grade. Indications for surgery were chronic colitis in 19 patients and dysplasia in one patient and two patients underwent surgery due to acute colitis.

The patients excluded due to logistic reasons did not differ concerning age, blood tests, or disease severity. Of 12 excluded patients, 7 were males. Median age was 42 (21–67). Median laboratory tests at surgery were haemoglobin 132 (107–155) g/L, C-reactive protein (CRP) 13 (1–190) mg/L, and albumin 30 (26–40) g/L. Symptom duration was 15 (3–30) years. Total Mayo-score was median 7 (4–11). Eight patients in the excluded group were on oral steroids and one was on locally administrated steroids. Six had oral 5-ASA and one had 5-ASA locally. One patient was on immunomodulating therapy.

Control tissues were obtained from patients undergoing elective resection of the sigmoid colon and right-sided hemicolectomy due to repeated sigmoid volvulus (*n* = 2) or colonic cancer (*n* = 8), respectively. Collection of control tissues was sampled as far as possible (minimum 10 cm) from the tumor.

### 2.2. Tissue Specimens

Resected colonic tissue was immediately embedded in a plastic bag in the operating theatre, covered with crushed ice, and transported within 45 minutes to the Department of Pathology. Tissue specimens were collected according to a standardized protocol from each colonic and rectal area; from each patient, 5-6 (rectal resection) to 15–20 separate (colectomy) samples were analysed. In addition, specimens from selected areas were collected according to the pathologist judgment. The specimens were fixed in formalin and embedded in paraffin.

**Figure 2:**
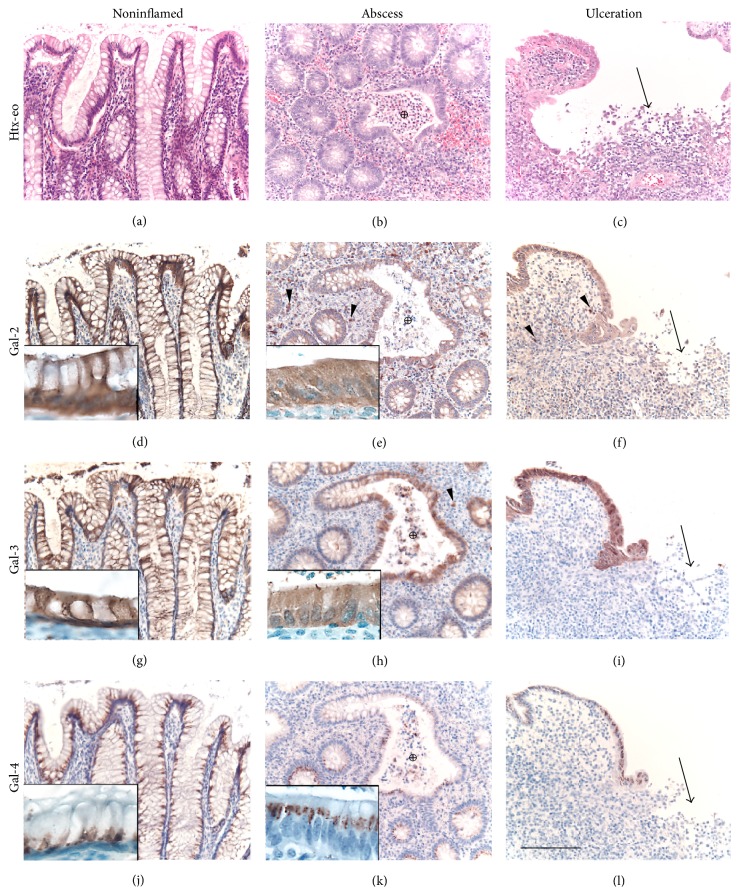
Immunostaining of galectin-2–galectin-4 in UC colon. Haematoxylin-eosin staining of noninflamed colon (a), ulcerative colitis with an abscess (marked *⨁* in (b)), and an ulceration (arrow in (c)) obtained from patient No 53 in Table 1. The noninflamed epithelium (a) contains cells filled with mucous and the crypts have a normal architecture. Immunohistochemical staining of serial sections from the same specimen for galectin-2, galectin-3, and galectin-4 is shown in rows 2 to 4, respectively. Galectins are predominantly expressed in the colonic epithelium. The inserts illustrate that galectin-2 and galectin-3 are homogeneously distributed in the cytoplasm while galectin-4 has a distinct perinuclear distribution most easily seen in (k) where mucus droplets are less abundant. In addition, a low number of inflammatory cells express a strong staining for galectin-2 and galectin-3, but not galectin-4, and this is illustrated for galectin-2 and galectin-3 by arrowheads in panels (e), (f), and (h). Bar = 200 *μ*m.

Paraffin sections of full wall colon tissue samples were obtained and stained with haematoxylin-eosin. The inflammatory activity for each patient was graded, according to suggested criteria for grading disease activity in UC [17], as mild (cryptitis), moderate (crypt abscesses), or severe (ulcerations) illustrated in Figure 2. All specimens were coded and analysed in a blinded fashion by two investigators (Johan Mölne and Mattias Block). The clinical course of each patient as well as the final histopathological evaluation based on all biopsies from each specimen was unknown to the investigators when they were evaluating the inflammatory grade and galectin expression in each tissue slide.

### 2.3. Anti-Galectin Antibodies

Polyclonal antisera were raised in rabbits and characterized as described for anti-rat galectin-1 (diluted 1 : 800) [35–37], anti-human galectin-2 (1 : 600) [38], and anti-rat galectin-4 (1 : 50000) [39, 40]. A commercially available rat monoclonal anti-mouse galectin-3 (anti-Mac-2, clone 3/38) (1 : 500) [41] has been used extensively by us [39] and others.

### 2.4. Immunohistochemistry

The EnVision Flex High pH (Link) detection kit (Dako K8000, Copenhagen, Denmark) was used. The most important steps were as follows. Consecutive series of paraffin sections were produced at a 4 *μ*m constant thickness setting. Antigen retrieval was done in tris/EDTA buffer, pH 9 (Dako K8004), by microwave oven heating and endogenous peroxidase activity was blocked by immersion in peroxidase-blocking solution (Dako K8000) for 5 minutes at RT. Immunostaining was performed in a computer-assisted Autostainer Plus processor (Dako). Incubation time for primary antibodies was 30 minutes at RT, terminated by repeated washing, followed by incubation with a dextran polymer conjugated with secondary antibodies and horseradish peroxidase (HRP) for another 30 minutes. Slides were transferred to fresh hydrogen peroxide plus 3-3-diaminobenzidine tetrahydrochloride (DAB) solutions for 4 minutes. Finally, slides were stained with Mayer's haematoxylin and permanently mounted under cover slips. Omitting or replacing the primary antibodies with irrelevant antibodies produced negative controls. Optimal primary antibody dilutions were defined by staining normal colon using serial dilutions of each antibody.

### 2.5. Grading of Inflammation and Immunohistochemical Labelling

**Figure 3:**
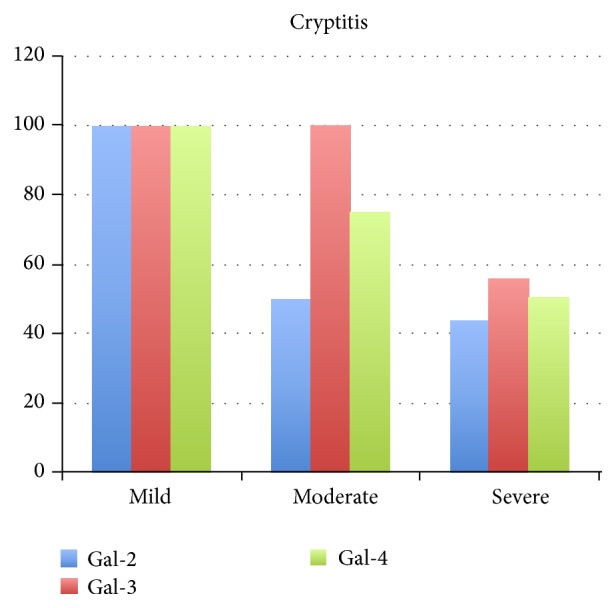
Summarized epithelial galectin-2, galectin-3, and galectin-4 expression in areas of cryptitis for the different individuals grouped according to the inflammatory grade (mild, moderate, and severe). Figures on the *y*-axis represent mean percentage of epithelial cell staining intensity; see Table 2.

Intestinal tissue specimens from each patient, 5-6 (rectal resection) to 15–20 separate (colectomy), were studied. Galectin-1, galectin-2, galectin-3, and galectin-4 expressions were examined in 3 representative tissue blocks from each patient. The epithelial cell galectin staining intensity was recorded in epithelia with cryptitis and crypt abscess and in areas adjacent to ulcerations as well as in noninflamed areas as illustrated in Figure 2. The intensity of immunoperoxidase (IP) staining was recorded on a 4-level scale: negative: 0, trace amounts: 1, weakly positive: 2, and strongly positive: 3. If galectin expression showed a focal pattern this was assigned as (f). Representative tissue sections illustrating galectin-2–galectin-4 expression in noninflamed colon tissue and IBD tissue with abscesses and ulcerations are shown in Figure 3. Leukocytes were identified by morphology (neutrophil and eosinophil granulocytes) or identified by monoclonal antibodies using CD3 (Dako, N1617) for T-cells, CD68 (Dako, N1576) for macrophages, and CD138 (Dako, IR642) for plasma cells.

## 3. Results

### 3.1. Epithelial Galectin Expression in Control Colon Tissue

Galectin-1 in normal colon epithelial cells was negative except in a few cases showing a focal, minimal staining. This is in accordance with earlier studies on normal human colon [42] and stomach [43]. In nonepithelial cells (fibroblasts, endothelium, and smooth muscle), galectin-1 was weakly expressed, as described before [44].

Galectin-2, galectin-3, and galectin-4 were all strongly positive as exemplified in Figures 2(d), 2(g), and 2(j), respectively, and did not show any individual variation (Table 2).

Galectin-2 staining was localised to the entire cytoplasm of the cells but mucus droplets were negative (Figure 2(d)). An increased staining was seen on the epithelial cell apical membrane (Figure 2(d), insert).

Galectin-3 staining was localised to the entire cytoplasm of the cell but mucus droplets were negative (Figure 2(g)). There was a gradient of galectin-3 staining intensity with strong surface epithelial expression and diminishing expression in crypts.

Galectin-4 in the control group showed, like galectin-2 and galectin-3, a strong expression pattern in all cases. However, compared to galectin-2 and galectin-3, labelling was different with a localised supranuclear distribution (Figures 2(j), insert, and  2(k)) and no staining was present in the remaining cytosol or plasma membrane.

### 3.2. Epithelial Galectin Expression in UC Colon (Study Group)

*Galectin-1*. Most of the UC patients, as well as the control individuals, lacked galectin-1 expression in the epithelial cells (not shown). Only a few cases showed a weak focal epithelial cell staining.

*Galectin-2*. In patients with mild inflammatory activity, the epithelial cell galectin-2 expression was identical to that seen in control individuals (Table 2). In those with moderate activity, most patients had a strong galectin-2 expression but some patients with severe activity showed a reduced-to-minimal epithelial expression in all 3 slides (Figures 2(e) and 2(f) and Table 2). Due to a reduced amount of mucus the entire cytoplasm was galectin-2 positive in areas of inflammation (Figures 2(e) and 2(f)). 

*Galectin-3*. The colon epithelium showed the same gradient of galectin-3 staining intensity with strong surface expression and diminishing expression in crypts, as in the control group (Figure 2(g)). In inflamed areas, staining was seen in the whole cytoplasm, as for galectin-2 (Figures 2(h) and 2(i)). A reduced expression of galectin-3 in some individuals with increased inflammatory activity was observed but the majority of patients expressed normal levels in severely inflamed specimens (Table 2). 

*Galectin-4*. A decreased galectin-4 expression in several patients with severe inflammatory activity (Figures 2(k) and 2(l) and Table 2) was observed but there was a great interindividual variation. The supranuclear distribution was seen also in inflamed epithelium (Figure 2(k) insert).

### 3.3. Individual Changes in Epithelial Cell Galectin Expression

In addition to Table 2, the epithelial galectin expression in areas with cryptitis, for the different individuals grouped according to the inflammatory grade (mild, moderate, and severe), is summarized in Figure 3. This shows that the galectin expression in the epithelial cells is decreased related to the severity of inflammation. However, when comparing individual patients, changes in epithelial cell galectin expression varied considerably between individual patients reflecting an individual pattern rather than a general reduction in galectin expression (Table 2). In the two cases with mild inflammation there was no change in galectin expression related to the inflammatory grade. Of the 4 cases with moderate inflammatory grade, 2 cases (No 48 and No 50) showed a galectin expression in the normal epithelial cells that was identical to that of the healthy controls (all positive) and no change in their expression was found irrespectively of inflammatory grade. Patients 1 and 14 had very small changes in galectin expression. The 16 patients with severe inflammation showed different pattern of changes in their epithelial cell galectin-2, galectin-3, and galectin-4 expression. These heterogenic patterns can be explained by the focality of UC disease and also individual factors among the patients as shown in Figure 1, where a patient with severe inflammation showed cryptitis, abscess, and ulceration in the same specimen.

### 3.4. Galectin Expression in Inflammatory Cells

In control colon tissue, inflammatory cells were seen in the lamina propria. Individual cells showed a labelling intensity comparable to inflammatory cells in the study. As expected, the number of inflammatory cells in the control tissue was considerably lower than in the study group.

The majority of inflammatory cells found in the intestinal wall in the study group were macrophages, lymphocytes, and plasma cells identified by morphology and specific CD markers. As expected, neutrophilic granulocytes were seen in areas of cryptitis, crypt abscesses, and ulcerations but were otherwise sparse. In the mucosa, approximately 50% of the leukocytes were plasma cells, 25% macrophages, and 25% T-cells. In the submucosa, the dominating cell type was macrophages, while only occasional and focal infiltrates of lymphocytes and plasma cells were seen.

Galectin-1 and galectin-4 were not present in any inflammatory cells.

Galectin-2 was strongly expressed in inflammatory cells in the majority of the patients irrespectively of the intestinal inflammatory grade. The labelling intensity was comparable to the galectin-2 expression in epithelial cells in very few inflammatory cells (<2%), while about 50% of the cells showed a weak expression (Figures 2(e) and 2(f)). Almost all of the strongly galectin-2 positive cells were identified as macrophages. However, in total, only a minority (10–20%) of the macrophages were positive. The majority of plasma cells (75–100%) were weakly positive as well as few (<5%) of the T-cells.

Galectin-3 expression in the inflammatory cell infiltrates varied between individual patients. Both negative and positive as well as trace amount of staining were noted for patients irrespectively of the intestinal inflammatory grade. For patients expressing galectin-3 in inflammatory cells, the expression was weaker compared to galectin-2, with an overall strong expression in <1% of the inflammatory cells and a weak expression in 10–15%. The majority of the positive inflammatory cells were macrophages and 20–30% of macrophages were positive for galectin-3. About 10% of the plasma cells and <1% of T-cells were positive for galectin-3 (Figure 2(h)).

## 4. Discussion

The UC tissues analysed in this study were obtained after bowel resection. This permits evaluation of the entire intestinal wall as well as several different intestinal regions compared to a limited number of small biopsies collected during endoscopy [32, 33]. Another difference compared to previous studies is that the surgical specimens were obtained from patients being at the end of the road regarding medical treatment of their disease and therefore representing, on average, a later state of the disease. However, there was still considerable heterogeneity regarding the degree of inflammation within a single intestine (exemplified in Figure 1) and also between individual patients. The control tissue in this study was from both the sigmoid area (*n* = 2) and ascending area of colon (*n* = 8) and no difference in galectin pattern was seen irrespective of which anatomical part the samples were collected from, nor was there any difference in galectin pattern depending on the anatomical localisation found in the UC study patients (Table 1). Therefore, it is not likely that the differences in galectin expression depend on the anatomical localisation of the tissue sections.

The galectin expression did not differ considerably between the colon epithelium in the control group and that of epithelial cells having a normal histological appearance in the UC study group. When the galectin expression in areas with cryptitis was summarized for each inflammatory grade (Figure 3) it seems that a decrease in epithelial galectin expression is correlated to inflammation. However, since the expression pattern for each individual patient was very complex (Table 2) we argue that there was no clear-cut correlation between the expressions of galectin-2, galectin-3, and galectin-4 in the colonic epithelium and the inflammatory activity in our study group of consecutive patients. This is in contrast to some reports that suggest decreased galectin expression in conjunction with intestinal inflammation [18, 20, 32]. There may be several explanations for this discrepancy. The evaluation of a certain antigen(s) in tissues/cells involves many technical aspects of the methodologies used that affect the quality of analysis. Immunohistochemistry has a well-known variation depending on the techniques for tissue handling and antibody reagents used. Molecular analysis strategies using homogenized biopsies also have limitations due to sampling errors. For example, galectin-3 has been quantified using mRNA reverse transcription technique [20]. However, there is usually a patchy appearance of the intestinal inflammatory process both macroscopically and, especially, at the microscopic level, with differences in intensity between different cryptitis, abscess, and ulceration areas (Figures 1 and 2). Therefore, quantification of whole biopsies collected by endoscopy has to be carefully evaluated regarding representativeness of tissue specimens analysed. Furthermore, changes in galectin expression in biopsies will be due to either loss of epithelial cells, reduced expression in individual cells, the amount of infiltrating inflammatory cells, or a combination of these factors. Many of the individual patients express a stable amount of galectins in their epithelial cells independently of the degree of inflammation (Table 2). Therefore, results from previous studies indicating that a reduced galectin expression correlates with inflammation [20, 32] may be due to lack of epithelial cells due to tissue damage as well as sampling error of small biopsies and not to a specific downregulation of epithelial galectin biosynthesis. Furthermore, to increase the number of individual cases analysed in this investigation will most likely not result in a statistical significant correlation between galectin expression and inflammation due to the great heterogeneity of epithelial cell galectin expression both within and between individual cases as shown in Table 2. Even if a statistical significant correlation should be obtained, the biological significance of this must be highly questioned.

The tissue localisation of galectin-1–galectin-4 found here agreed in general with previous immunohistochemical studies of human and mouse intestines. In addition, we also found some features not described before. Galectin-1, one of the most studied galectins, has typically not been found in human epithelial cells [42, 43], and this was also the case here. This is in contrast to mouse where galectin-1 is expressed in the intestinal epithelial cells [45, 46] but also shows a strain variation [46]. Variable expression of galectin-1 has been found in human nonepithelial tissue like muscle, lymphocytes, and fibroblasts [42] but here no or low expression was found. However, this does not rule out a role in colitis, as galectin-1 can bind both epithelial cells and lymphocyte glycans, resulting in apoptosis and other cell regulations [47].

Galectin-2 is the closest compared to galectin-1 but much less studied in mammals. Similar to galectin-1, it is a noncovalent dimer, but unlike galectin-1 it has characteristic localisation to the gastrointestinal tract and it has a different carbohydrate-binding specificity, seemingly adapted to intestinal glycans [18, 19]. Thus, it can be regarded as an intestinal paralogue of galectin-1. Here we found high expression of galectin-2 in colon epithelial cells, as reported before, but also the novel observation of high expression in some submucosal macrophages, suggesting an immunoregulatory role. Added galectin-2 can support epithelial wound healing and suppress lamina propria T-cells to ameliorate experimental colitis in mice [18].

Galectin-3, the other most studied galectin, was abundant in epithelial cells and also in some macrophages, in agreement with previous studies [20, 24]. Galectin-3 behaves mainly as a proinflammatory protein, and studies using null mutant mice support a rate-limiting role in chronic inflammation with fibrosis in many tissues. In a mice colitis experimental model, intraperitoneally administrated galectin-3 reduced inflammation [48].

In contrast to galectin-2 and galectin-3, which were distributed in the entire cytoplasm, galectin-4 had specific supranuclear localisation presumed to be in the Golgi network. To our knowledge, this has not been reported before. Galectin-4 is expressed only in the digestive tract and restricted to epithelial cells. Previous studies have showed that there is no significant difference in the expression of galectin-4 in epithelial cells from inflamed colon versus controls [18] which is confirmed by this study.

Information regarding galectin expression in the infiltrating inflammatory cells in IBD intestine is very limited. In the macrophages, we found a strong expression of galectin-2 and a weak expression of galectin-3 with a significant interindividual variability, while staining for galectin-1 and galectin-4 was completely negative. There was no increase in the expression of galectin-2 and galectin-3 in the inflammatory infiltrates in the UC study group compared to the expression in inflammatory cells in controls. Reports on galectin expression in intestinal inflammatory cells have, to our knowledge, only been reported for galectin-3. A study of patients with ileal pouch-anal anastomosis [49] revealed a significant decrease in the galectin-3 staining index in subepithelial macrophages in patients with chronic or recurrent acute pouchitis compared to the noninflamed controls. A reduced expression of galectin-3 has been found in intestinal macrophages of patients with Crohn's disease but not in UC patients [50]. The observation of a high specific expression of galectin-2 in submucosal macrophages has not been reported earlier. Galectin-2 is evolutionary and structurally most related to galectin-1, but it has a tissue expression and carbohydrate-binding specificity more adapted to glycan structures found in intestinal epithelial cells (galactosides with blood group determinants) [47] and does not bind sialylated glycans as found in serum glycoproteins bound by galectin-1 [51].

## 5. Conclusions

The findings that several UC patients did not show any changes in colon epithelial cell galectin expression while others showed an individual specific change not correlated to the inflammatory grade indicate that the variation in epithelial galectin expression may not be related primarily to the inflammatory grade but rather to the focal presentation of the disease as well as individual factors not defined at present.

## Figures and Tables

**Table 1:** Clinical data of patients with UC included in the study. Study patients are grouped according to tissue inflammation degree as mild (cryptitis, *n* = 2), moderate (crypt abscesses, *n* = 4), and severe (ulcerations, *n* = 16) as described in Section 2. In addition, control colon tissues (*n* = 10) obtained from patients with volvulus and colon cancer were studied.

No^1^	Gender/age^2^	Performed procedure^3^	Disease duration^4^	Medical therapy SS/SL/AS/AL/IM^5^	Mayo-score^6^	CRP mg/L^7^	Infl-grade^8^
18	M/49	C	19	−/−/+/−/−	6	20	Mild
19	F/49	RR	21	−/−/−/−/−	10	5	Mild

1	M/29	C	3	+/−/+/−/+	7	5	Moderate
14	M/65	RR	10	−/−/+/−/−	10	5	Moderate
48	F/36	C	4	−/−/+/−/+	10	4	Moderate
50	F/38	C	6	−/−/+/−/+	5	9	Moderate

2	F/49	C	21	+/+/−/−/−	5	28	Severe
3^9^	F/22	C	7	+/+/−/−/−	10	5	Severe
4	F/53	C	2	+/+/+/−/−	9	5	Severe
5	F/28	RR	6	−/−/−/−/−	9	5	Severe
8	F/52	RR	25	−/−/−/−/−		5	Severe
11	F/38	C	21	+/−/+/−/+	8	44	Severe
13	F/27	RR	5	+/−/−/−/−	10	5	Severe
15	F/62	PC	8	+/−/+/+/−	7	5	Severe
17	F/33	C	7	+/+/+/+/−	8	160	Severe
22	M/42	C	14	+/−/−/+/−	11	5	Severe
26	F/39	RR	3	−/−/−/−/−	8	5	Severe
28^9^	M/24	C	9	+/−/+/−/−	11	22	Severe
29	M/20	C	7	+/+/+/−/+	6	18	Severe
40	M/35	RR	6	+/+/−/−/+	6	1	Severe
52	M/41	PC	5	−/−/−/−/+	11	2	Severe
53	F/27	C	17	−/−/−/−/+	10	10	Severe

^1^No = patient study number in IBD biobank.

^2^M = male. F = female. Age = years.

^3^C = colectomy. PC = proctocolectomy. RR = rectum resection.

^4^Disease duration = years.

^5^SS = steroids systemic. SL = steroids local. AS = azathioprine systemic. AL = azathioprine local. IM = immunomodulation.

^6^Mayo-score = clinical evaluation of the severity of the patient's disease.

^7^CRP = C-reactive protein.

^8^Infl-grade = total histopathological evaluation of the patients disease.

^9^Acute surgery.

**Table 2:** Galectin expression in colon epithelial cells in patients with UC. Study patients are grouped according to degree of tissue inflammation (see Table 1) and normal controls (*n* = 10). Epithelial cell staining intensity for individual anti-galectin antibodies was graded as 0 to 3. Galectin expression is shown for the epithelial cells present in the uninflamed, cryptitis, abscess, and ulceration areas. Areas not applicable (i.e., ulceration in mild inflammation cases) are marked with —. Staining for galectin-1 was negative in the epithelial cells of both controls and UC cases and is not listed.

Patient number	Inflammatory grade^*∗*^	Epithelial cell galectin expression^#^											
		Noninflamed area			Cryptitis			Abscess			Ulceration		
		Gal-2	Gal-3	Gal-4	Gal-2	Gal-3	Gal-4	Gal-2	Gal-3	Gal-4	Gal-2	Gal-3	Gal-4
18	Mild	3^†^	3	3	3	3	3	—^¶^	—	—	—	—	—
19	Mild	3	3	3	3	3	3	—	—	—	—	—	—

1	Moderate	2	3	1	2	3	1	2	3	1	—	—	—
14	Moderate	3	3	3	2	3	3	2	3	3	—	—	—
48	Moderate	3	3	3	3	3	3	3	3	3	—	—	—
50	Moderate	3	3	3	3	3	3	3	3	3	—	—	—

2	Severe	1	3	2	3	1	1	3	1	0	3	3	0
3	Severe	3	3	2	2	1	1	1	1	1	1	3	2
4	Severe	3	3	3	1	1	3	1	3	2	2	2	3
5	Severe	2 (f)^‡^	3	3	1	1	3	1	3	3	1	2	3
8	Severe	3	3	3	2	3	2	—	—	—	1	1	1
11	Severe	3	3	3	2	3	2	2	3	2	2	3	2
13	Severe	2	3	2	—	—	—	—	—	—	1	1	2
15	Severe	3	3	3	3	3	2	3	3	2	3	3	3 (f)
17	Severe	3	3	3	2	3	3	2	3	3	3	3	3
22	Severe	3	3	3	3	3	3	3	3	3	2	3	2
26	Severe	3	3	3	1	3	3	1	3	3	3 (f)	3	2
28	Severe	1	3	1	3	3 (f)	3	3 (f)	3 (f)	3	3 (f)	3 (f)	3
29	Severe	3	3	3	3	2	3	3	2	3	2	2	2
40	Severe	3	3	2	—	—	—	2	2	3	3	3	3
52	Severe	3	3	1	3	3	1	—	—	—	3	3	1
53	Severe	3	3	3	3	3	3	3	3	3	3	3	3

Controls *n* = 10	Normal	3	3	3	—	—	—	—	—	—	—	—	—

^*∗*^Histopathological classification of inflammatory activity.

^#^Galectin-2, galectin-3, and galectin-4 expression in epithelial cells present in the area.

^†^Galectin expression is graded according to a 4-level scale as follows: 0 = negative, 1 = trace, 2 = weak expression, and 3 = strong expression.

^¶^Empty fields (not marked with 0–3) mean no presence of cryptitis, abscess, or ulceration.

^‡^Focal distribution in some cells in the area.
